# Frequency of Adverse Events in the Placebo Arms of COVID-19 Vaccine Trials: A Systematic Review and Meta-analysis

**DOI:** 10.1001/jamanetworkopen.2021.43955

**Published:** 2022-01-18

**Authors:** Julia W. Haas, Friederike L. Bender, Sarah Ballou, John M. Kelley, Marcel Wilhelm, Franklin G. Miller, Winfried Rief, Ted J. Kaptchuk

**Affiliations:** 1 Program in Placebo Studies, Beth Israel Deaconess Medical Center, Harvard Medical School, Boston, Massachusetts; 2 Division of Gastroenterology, Beth Israel Deaconess Medical Center, Harvard Medical School, Boston, Massachusetts; 3 Department of Clinical Psychology and Psychotherapy, Philipps University Marburg, Marburg, Germany; 4 Department of Psychology, Endicott College, Beverly, Massachusetts; 5 Weill Cornell Medical College, New York, New York

## Abstract

**Importance:**

Adverse events (AEs) after placebo treatment are common in randomized clinical drug trials. Systematic evidence regarding these nocebo responses in vaccine trials is important for COVID-19 vaccination worldwide especially because concern about AEs is reported to be a reason for vaccination hesitancy.

**Objective:**

To compare the frequencies of AEs reported in the placebo groups of COVID-19 vaccine trials with those reported in the vaccine groups.

**Data Sources:**

For this systematic review and meta-analysis, the Medline (PubMed) and Cochrane Central Register of Controlled Trials (CENTRAL) databases were searched systematically using medical subheading terms and free-text keywords for trials of COVID-19 vaccines published up to July 14, 2021.

**Study Selection:**

Randomized clinical trials of COVID-19 vaccines that investigated adults aged 16 years or older were selected if they assessed solicited AEs within 7 days of injection, included an inert placebo arm, and provided AE reports for both the vaccine and placebo groups separately. Full texts were reviewed for eligibility by 2 independent reviewers.

**Data Extraction and Synthesis:**

Data extraction and quality assessment were performed independently by 2 reviewers, adhering to the Preferred Reporting Items for Systematic Reviews and Meta-analyses (PRISMA) guideline and using the Cochrane risk-of-bias tool. Meta-analyses were based on random-effects models.

**Main Outcomes and Measures:**

The primary outcomes were the proportions of placebo recipients reporting overall, systemic, and local (injection-site) AEs as well as logarithmic odds ratios (ORs) to evaluate group differences. Outcomes were tested for significance using *z* tests with 95% CIs.

**Results:**

Twelve articles with AE reports for 45 380 participants (22 578 placebo recipients and 22 802 vaccine recipients) were analyzed. After the first dose, 35.2% (95% CI, 26.7%-43.7%) of placebo recipients experienced systemic AEs, with headache (19.3%; 95% CI, 13.6%-25.1%) and fatigue (16.7%; 95% CI, 9.8%-23.6%) being most common. After the second dose, 31.8% (95% CI, 28.7%-35.0%) of placebo recipients reported systemic AEs. The ratio between placebo and vaccine arms showed that nocebo responses accounted for 76.0% of systemic AEs after the first COVID-19 vaccine dose and for 51.8% after the second dose. Significantly more vaccine recipients reported AEs, but the group difference for systemic AEs was small after the first dose (OR, −0.47; 95% CI, −0.54 to −0.40; *P* < .001; standardized mean difference, −0.26; 95% CI, −0.30 to −0.22) and large after the second dose (OR, −1.36; 95% CI, −1.86 to −0.86; *P* < .001; standardized mean difference, −0.75; 95% CI, −1.03 to −0.47).

**Conclusions and Relevance:**

In this systematic review and meta-analysis, significantly more AEs were reported in vaccine groups compared with placebo groups, but the rates of reported AEs in the placebo arms were still substantial. Public vaccination programs should consider these high rates of AEs in placebo arms.

## Key Points

**Question:**

What was the frequency of adverse events (AEs) in the placebo groups of COVID-19 vaccine trials?

**Findings:**

In this systematic review and meta-analysis of 12 articles including AE reports for 45 380 trial participants, systemic AEs were experienced by 35% of placebo recipients after the first dose and 32% after the second. Significantly more AEs were reported in the vaccine groups, but AEs in placebo arms (“nocebo responses”) accounted for 76% of systemic AEs after the first COVID-19 vaccine dose and 52% after the second dose.

**Meaning:**

This study found that the rate of nocebo responses in placebo arms of COVID-19 vaccine trials was substantial; this finding should be considered in public vaccination programs.

## Teaser

This systematic review and meta-analysis investigates the frequency of adverse events in placebo groups compared with that in vaccine groups in COVID-19 vaccine trials.

## Introduction

The ongoing COVID-19 pandemic has caused more than 5 million deaths worldwide^1^ and led to tremendous physical, mental, and economic hardships. Several vaccines have been developed and tested within remarkably short periods. Currently, public vaccination programs have already achieved success in reducing the numbers of new infections in several countries. However, a substantial proportion of the population (internationally estimated at approximately 20%) intends to refuse vaccination.^2,3,4^ In 2019, before the COVID-19 pandemic, the World Health Organization claimed vaccination hesitancy as a global health threat^5^; this threat is particularly salient in the case of COVID-19. Counteracting the underlying motivations for vaccination hesitancy is therefore crucial to overcome this worldwide crisis.

Although the reasons for vaccination hesitancy are diverse and complex, concerns about potential adverse events (AEs) from the COVID-19 vaccines seem to be a major factor.^6^ According to a global survey from January 2021, 47% of respondents were worried about AEs from a COVID-19 vaccine.^7^ With regard to influenza vaccination, there is broad evidence of an association between concerns about AEs and vaccination refusal.^8,9,10,11,12^ Several systematic reviews of randomized clinical drug trials have demonstrated that the occurrence of AEs can also be substantial in placebo arms.^13^ Adverse events seemingly elicited by placebos are often called *nocebo responses*^14^ and are thought to be caused by misattribution of routine background symptoms,^15^ anxiety,^16^ and expectations of AEs.^17,18^ Emerging research has shown that informing patients about nocebo responses^19,20^ and providing a positive framing of potential AEs^21,22,23,24^ may be associated with reduced AE-related anxiety and nocebo responses. Although nocebo phenomena have been investigated in many contexts involving medication,^18,25,26,27,28^ evidence of their influence in vaccination remains scarce. However, a recent meta-analysis suggested that a significant proportion of placebo recipients in influenza vaccine trials experienced systemic AEs, such as headache or fatigue, owing to nocebo responses.^29^

Researchers of nocebo response have called attention to ubiquitous nocebo responses in COVID-19 vaccination,^30,31^ but systematic quantification is needed. The current systematic review and meta-analysis aimed to assess the frequencies of AEs reported in the placebo groups of COVID-19 vaccine trials and compare them with the frequencies of AEs reported in the vaccine groups.

## Methods

This systematic review and meta-analysis followed the Preferred Reporting Items for Systematic Reviews and Meta-analyses (PRISMA) reporting guideline.^32^ The study protocol has been registered in PROSPERO (CRD42021256905).

### Eligibility Criteria

We included randomized clinical trials of experimental COVID-19 vaccines that investigated adults aged 16 years or older and were published in English. Studies were eligible if they assessed solicited AEs within 7 days of injection, included an inert placebo arm (eg, saline), and provided AE reports for both the vaccine and the placebo groups separately. Studies were excluded if the applied placebo contained any active ingredient that could have caused specific AEs (eg, an adjuvant) or if risk of bias was judged as unknown.

### Search Strategy

A systematic literature search of studies published up to July 14, 2021, was conducted across the Medline database (PubMed) and the Cochrane Central Register of Controlled Trials (CENTRAL). These databases were searched for (1) medical subheading terms and (2) free-text keywords (eAppendix in the Supplement).

### Study Selection and Assessment

After titles and abstracts were screened for initial eligibility by J.W.H., 2 of us (J.W.H. and F.L.B.) independently reviewed the full texts of potentially eligible articles for inclusion and exclusion criteria. Data extraction and quality assessment were performed independently by J.W.H and F.L.B. Any discrepancies were resolved through discussion and, if necessary, evaluation by a third team member (S.B.).

Only AEs in terms of solicited reactogenicity symptoms were derived. Long-term observations or follow-up data were not considered as outcomes in the meta-analysis, and neither were serious AEs. Because all included trials investigated 2-dose schemes of vaccination, data on AEs after the first dose and on those after the second dose were extracted separately. If data on AEs were not provided in sufficient detail in the published material, the authors were emailed with a request. For studies that investigated different dosing schemes within 1 trial, only 1 of the vaccine groups was selected for comparison with the placebo group. To ensure practical relevance, we always selected the dose that was chosen for clinical application or described as the preferred dose for further investigation. The quality of the methods and the internal validity of each included study were rated based on predefined criteria covering the 7 categories of the Cochrane risk-of-bias tool.^33^

### Statistical Analysis

Data management and statistical analyses were performed using Microsoft Excel (Microsoft Corporation)^34^ and the R-based software JASP, version 0.14.1.0 (University of Amsterdam).^35^ Adverse events in different trials were summarized into homogeneous categories, combining AEs that were considered to measure the same symptom (eg, arthralgia and joint pain) or represented symptoms closely related to each other (eg, nausea and vomiting). Overall AE categories were any AE, any local (injection-site) AE, and any systemic AE. Adverse event categories had to be reported in at least 4 trials to be included in the meta-analysis. In addition to the analysis of the first and second doses separately, data on both doses were merged by calculating means if studies did not provide overall AE data. This may have underestimated true AE frequencies and was therefore considered to have increased risk of bias.

To examine AE frequencies in the placebo groups, the proportions of participants experiencing the respective symptoms were calculated. To compare AE frequencies between the groups, logarithmic odds ratios (log ORs) were calculated. Log ORs were also calculated to compare frequencies of AEs after the first and second doses within the groups. Significance was set at 2-sided *P* < .05. Meta-analyses were performed based on a restricted maximum-likelihood model (random effects) including 95% CIs. For the categories of any systemic AE and any local AE, ratios between the pooled placebo and vaccine AE proportions were used to calculate the percentage of AEs that were accounted for by nocebo responses. The pooled log ORs were tested for significant divergence from 0 using *z* tests; for the presence of adverse events, no statistical tests were done because for proportions, the null hypothesis (ie, that there were no cases in the population) could be rejected whenever a single case was reported. Heterogeneity was tested using *Q* tests and quantified using *I*^2^ tests. A variation in outcome (*I*^2^) greater than 50% was considered to derive from heterogeneity.^36^ Funnel plots were visually screened for asymmetry to detect publication bias,^37^ although publication bias was considered less relevant to a meta-analysis assessing nocebo responses than to one assessing the efficacy of a drug.^28^ To control for differences in the quality of the methods as identified by the risk-of-bias assessment, all analyses were rerun including the 3 heterogeneous risk-of-bias categories as factors. Owing to the small number of included trials, the results of these mixed-model analyses may be less reliable than those of the primary analyses and are therefore presented in eTable 2 in the Supplement.

Because data on AE severity grading were not reported in comparable detail by a sufficient number of trials to be analyzed meta-analytically, we descriptively explored the 2 largest trials that provided these data in detail.^38,39^ In these exploratory analyses, we calculated the proportions of severity grades in participants experiencing any AEs, any local AEs, and any systemic AEs after the first and second doses of placebo or vaccine separately.

## Results

### Search Results and Study Characteristics

**Figure 1.**
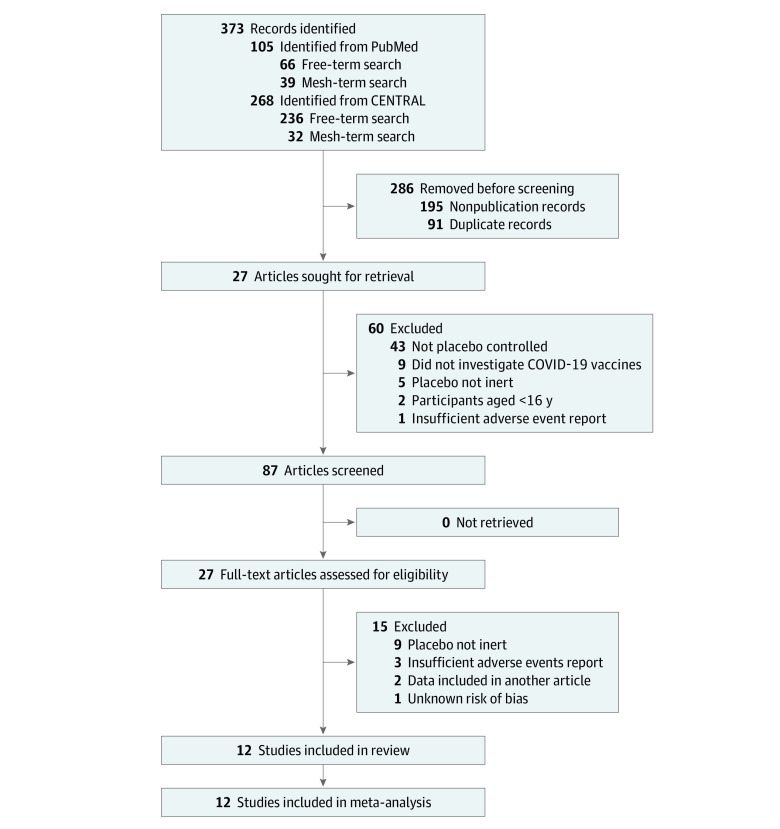
PRISMA Flow Diagram

Of 87 screened articles, 27 publications were included for full-text review (Figure 1). Twelve of these studies^38,39,40,41,42,43,44,45,46,47,48,49^ were included in our analyses (Table 1), resulting in a total of 45 380 participants; 22 578 placebo recipients and 23 817 active-vaccine recipients provided AE reports, but owing to multiple vaccine groups in some trials,^40,41,42,43,44,45,46^ only 22 802 of the vaccine recipients were included in our analyses. The numbers and percentages of AEs reported in the placebo groups and vaccine groups selected for comparison are provided in eTable 1 in the Supplement.

**Table 1.** Characteristics of the 12 Analyzed Randomized Clinical Trials of COVID-19 Vaccines

Study	Vaccine characteristics				Trial characteristics		
	Name of tested vaccine (manufacturer)	Manufacturing method	Adjuvant application	Days between doses	Clinical trial phase	Countries of assessment	Participants with assessment of adverse events, No.
Baden et al,^38^ 2020	mRNA-1273 (Moderna)	mRNA based	None	28	3	US	30 323
Chu et al,^43^ 2021	mRNA-1273 (Moderna)	mRNA based	None	28	2	US	599
Goepfert et al,^46^ 2021	CoV2 preS dTM (Sanofi Pasteur)	Protein based	Added to vaccine only	21	1-2	US	269
Heath et al,^39^ 2021	NVX-CoV2373 (Novavax)	Protein based	Added to vaccine only	21	3	UK	2714
Keech et al,^44^ 2020	NVX-CoV2373 (Novavax)	Protein based	Added to vaccine only	21	1-2	Australia	125
Li et al,^45^ 2021	BNT162b1 (BioNTech/Pfizer)	mRNA based	None	21	1	China	144
Madhi et al,^47^ 2021	ChAdOx1 nCoV-19/AZD1222 (AstraZeneca)	Vector based	None	28	1-2	South Africa	1920
Polack et al,^48^ 2020	BNT162b2 (BioNTech/Pfizer)	mRNA based	None	21	2-3	Argentina, Brazil, South Africa, US	8183
Richmond et al,^41^ 2021	SCB-2019 (Clover)	Protein based	Added to vaccine only	21	1	Australia	150
Sadoff et al,^42^ 2021	Ad26.COV2.S (Johnson & Johnson)	Vector based	None	56	1-2	Belgium, US	805
Shinde et al,^49^ 2021	NVX-CoV2373 (Novavax)	Protein based	Added to vaccine only	21	2a-b	South Africa	968
Walsh et al,^40^ 2020	BNT162b1 and BNT162b2 (BioNTech/Pfizer)	mRNA based	None	21	1	US	195

The risk-of-bias assessment revealed that the included publications were of high quality in general. In all included studies, the risk of bias was low with regard to randomization, blinding, and outcome assessment. However, the risk of bias was greater in several studies^40,41,42,43^ owing to the inclusion of sentinel participants (enrolled and evaluated before the other participants for safety purposes). One study^44^ reported a high dropout rate (16%) in the placebo group only. Another study^45^ provided AE data on the first and second doses within a 14-day period instead of 7 days. One study^50^ was excluded owing to an unknown risk of bias in the outcome assessment.

### Proportions of AEs

**Figure 2.**
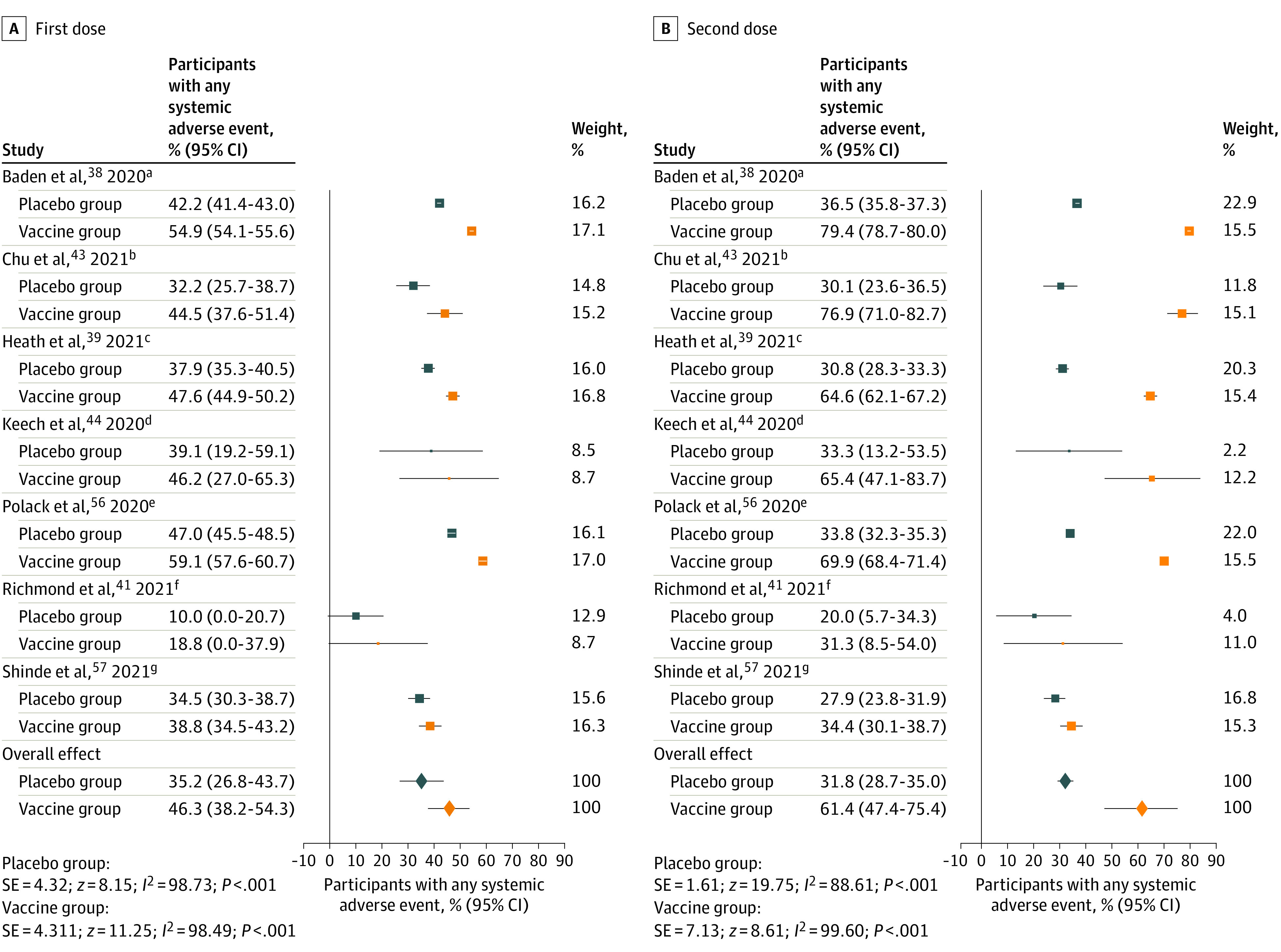
Forest Plots of Any Systemic Adverse Events After the First and Second Doses of the COVID-19 Vaccine or Placebo Random-effects pooled proportions are shown. Boxes represent the effect size of each study and whiskers, 95% CIs. Box size indicates the study’s weight in the analysis. Diamonds indicate pooled estimates, with whiskers indicating 95% CIs. Studies that did not provide data for the “any systemic adverse event” category were not included in these analyses but only in analyses on other adverse event categories. All placebos used were inert saline solutions. ^a^mRNA-1273 (Moderna, mRNA vaccine, phase 3 trial). Probability of being randomized to placebo group, 50%. ^b^mRNA-1273 (Moderna, mRNA vaccine, phase 2 trial). Vaccine group was selected for comparison in trials that investigated multiple dosing schemes (100 μg [high dose]). Probability of being randomized to placebo group, 33%. ^c^NVX-CoV2373 (Novavax, protein-based vaccine, phase 3 trial). Vaccine contained adjuvants. Probability of being randomized to placebo group, 50%. ^d^NVX-CoV2373 (Novavax, protein-based vaccine, phase 1-2 trial). Vaccine contained adjuvants. Vaccine group was selected for comparison in trials that investigated multiple dosing schemes (5 μg [low dose] + adjuvant). Probability of being randomized to placebo group, 20%. ^e^BNT162b2 (BioNTech/Pfizer, mRNA vaccine, phase 2-3 trial). Probability of being randomized to placebo group, 50%. ^f^SCB-2019 (Clover, protein-based vaccine, phase 1 trial). Vaccine contained adjuvants. Vaccine group was selected for comparison in trials that investigated multiple dosing schemes (30 μg [high dose] + adjuvant). Probability of being randomized to placebo group, 20%. ^g^NVX-CoV2373 (Novavax, protein-based vaccine, phase 2a-b trial); vaccine contained adjuvants. Probability of being randomized to placebo group, 50%.

The random-effects pooled proportion of placebo recipients reporting at least 1 systemic AE after the first dose was 35.2% (95% CI, 26.7%-43.7%); 16.2% (95% CI, 11.3%-21.1%) reported at least 1 local AE (Table 2). In comparison, patients treated with vaccines reported higher AE rates, with 46.3% (95% CI, 38.2%-54.3%) reporting at least 1 systemic AE and 66.7% (95% CI, 53.2%-80.3%) reporting at least 1 local AE. The ratios between the placebo and vaccine AE proportions suggest that after the first vaccine dose, nocebo responses accounted for 76.0% of systemic AEs (Figure 2) and 24.3% of local AEs.

**Table 2.** Coefficients of the Random-Effects Meta-analysis of Adverse Event Proportions in the Placebo Groups

Adverse event	Studies includedin analysis, No.	Proportion (95% CI)^a^	SE	*I*^2^, %
Any adverse event	5	0.306 (0.195 to 0.417)	0.06	94.1
**Any local adverse event**				
Overall	9	0.127 (0.084 to 0.171)	0.02	97.5
Dose 1	8	0.162 (0.113 to 0.211)	0.03	97.8
Dose 2	8	0.118 (0.066 to 0.171)	0.03	99.2
Pain				
Overall	10	0.100 (0.068 to 0.131)	0.02	95.2
Dose 1	10	0.100 (0.063 to 0.136)	0.02	96.6
Dose 2	10	0.088 (0.052 to 0.124)	0.02	98.2
Redness				
Overall	11	0.003 (0.001 to 0.005)	<0.01	91.3
Dose 1	11	0.003 (0.001 to 0.005)	<0.01	92.2
Dose 2	11	0.002 (<0.001 to 0.003)	<0.01	56.0
Swelling				
Overall	10	0.002 (0.001 to 0.005)	<0.01	89.3
Dose 1	10	0.003 (0.001 to 0.005)	<0.01	90.0
Dose 2	10	0.002 (0.001 to 0.004)	<0.01	89.1
Tenderness				
Overall	6	0.092 (0.043 to 0.140)	0.03	97.9
Dose 1	6	0.112 (0.054 to 0.170)	0.03	98.2
Dose 2	6	0.071 (0.030 to 0.113)	0.02	98.3
**Any systemic adverse event**				
Overall	9	0.298 (0.230 to 0.365)	0.04	98.0
Dose 1	8	0.352 (0.267 to 0.437)	0.04	98.7
Dose 2	8	0.318 (0.287 to 0.350)	0.02	88.6
Fever				
Overall	12	0.003 (0.001 to 0.005)	<0.01	81.5
Dose 1	12	0.004 (0.001 to 0.006)	<0.01	91.7
Dose 2	12	0.003 (0.001 to 0.005)	<0.01	47.1
Chills				
Overall	6	0.030 (0.012 to 0.049)	0.01	97.5
Dose 1	6	0.034 (0.014 to 0.054)	0.01	97.7
Dose 2	6	0.026 (0.009 to 0.044)	0.01	97.5
Fatigue				
Overall	10	0.159 (0.100 to 0.218)	0.03	99.4
Dose 1	10	0.167 (0.098 to 0.236)	0.04	99.7
Dose 2	10	0.149 (0.098 to 0.201)	0.03	99.2
Malaise				
Overall	5	0.078 (0.042 to 0.114)	0.02	75.7
Dose 1	5	0.080 (0.042 to 0.118)	0.02	78.0
Dose 2	5	0.069 (0.020 to 0.117)	0.03	95.8
Joint pain				
Overall	9	0.068 (0.047 to 0.089)	0.01	94.2
Dose 1	9	0.066 (0.038 to 0.094)	0.01	98.5
Dose 2	9	0.063 (0.044 to 0.082)	0.01	93.4
Muscle pain				
Overall	11	0.082 (0.055 to 0.110)	0.01	97.9
Dose 1	11	0.091 (0.060 to 0.121)	0.02	98.3
Dose 2	11	0.072 (0.047 to 0.097)	0.01	97.6
Headache				
Overall	11	0.184 (0.145 to 0.224)	0.02	95.5
Dose 1	11	0.193 (0.136 to 0.251)	0.03	98.1
Dose 2	11	0.162 (0.125 to 0.198)	0.02	95.2
Nausea and/or vomiting				
Overall	9	0.029 (0.010 to 0.049)	0.01	98.6
Dose 1	9	0.031 (0.010 to 0.052)	0.01	99.0
Dose 2	9	0.028 (0.008 to 0.047)	0.01	98.6
Diarrhea				
Overall	4	0.043 (0.005 to 0.081)	0.02	79.0
Dose 1	4	0.031 (0.001 to 0.078)	0.02	99.3
Dose 2	4	0.033 (0.001 to 0.070)	0.02	97.0

^a^ No statistical tests for the proportions of adverse events were done because the null hypothesis (ie, that there were no cases in the population) could be rejected whenever a single case was reported.

After the second dose, AE proportions in placebo groups were lower, with 31.8% (95% CI, 28.7%-35.0%) of participants reporting any systemic AEs and 11.8% (95% CI, 6.6%-17.1%) reporting any local AEs (Table 2). These differences in AE rates between the first and second doses within the placebo groups were statistically significant (any systemic AE: log OR, 0.33 [95% CI, 0.18-0.47]; SE, 0.08; *z*, 4.29; *P* < .001; any local AE: log OR, 0.22 [95% CI, 0.08-0.36]; SE, 0.07; *z*, 3.06; *P* = .002). However, AE proportions in the vaccine groups were greater after the second dose than after the first, with 61.4% (95% CI, 47.4%-75.4%) of participants reporting any systemic AEs and 72.8% (95% CI, 57.4%-88.2%) reporting any local AEs. The differences between the first and second doses within the vaccine groups were statistically significant for systemic AEs (log OR, −0.71 [95% CI, −1.16 to −0.26]; SE, 0.23; *z*, −3.09, *P* = .002) but not for local AEs (log OR, −0.29 [95% CI, −0.73 to 0.14]; SE, 0.22; *z*, −1.33; *P* = .18).

Thus, compared with the first dose, a larger difference in AE rates between the placebo groups and vaccine groups was found after the second dose (Figure 2). Nevertheless, ratios between AE proportions in the placebo and vaccine groups indicated that nocebo responses accounted for 51.8% of systemic and 16.2% of local AEs after the second dose. The most commonly reported AEs in the placebo groups were headache (first dose: 19.3% [95% CI, 13.6%-25.1%]; second dose: 16.2% [95% CI, 12.5%-19.8%]) and fatigue (first dose: 16.7% [95% CI, 9.8%-23.6%]; second dose: 14.9% [95% CI, 9.8%-20.1%]). Heterogeneity of the included studies was very high (*I*^2^≥50%) for most AE categories.

Mixed-model analyses indicated that the proportions of placebo recipients with AEs may have been higher when controlling for risk-of-bias variables, with 40.5% (95% CI, 32.9%-48.2%) of participants reporting at least 1 systemic AE after the first dose (eTable 2 in the Supplement). Funnel plots did not show asymmetry of data points, but heterogeneity was still very high for most AE categories.

### Effect Sizes of Group Differences

Most random-effects pooled log ORs were statistically significant, suggesting that AE rates were significantly higher in the vaccine groups compared with the placebo groups (Table 3). After the first dose, the pooled log OR for any systemic AE was −0.47 (95% CI, −0.54 to −0.40; *P* < .001), which is equivalent to a standardized mean difference of −0.26 (95% CI, −0.30 to −0.22). For any local AE after the first dose, the pooled log OR was −2.44 (95% CI, −3.21 to −1.66), which is equivalent to a standardized mean difference of −1.34 (95% CI, −1.77 to −0.92). Thus, the difference between placebo and vaccine recipients experiencing any local AE after the first dose equated to a large effect, whereas it equated to only a small effect for any systemic AE. Effect sizes were not significant for nausea and diarrhea, and they were small for fatigue, malaise, joint pain, and headache (Table 3), suggesting only minor differences between the placebo and vaccine groups in the experience of these AEs after the first dose.

**Table 3.** Coefficients of the Random-Effects Meta-analysis of Logarithmic Odds Ratios to Compare the Occurrence of Adverse Events in the Placebo and Vaccine Groups

Adverse event	Participants reporting adverse events, % (95% CI)^a^		Logarithmic odds ratio (95% CI)^b^	SE	*z*	*P* value	*I*^2^, %	Standardized mean difference (95% CI)
	Placebo groups	Vaccine groups						
Any adverse event	30.6 (19.5 to 41.7)	76.2 (55.2 to 97.2)	−2.33 (−3.65 to −1.02)	0.67	−3.48	<.001	97.71	−1.29 (−2.01 to −0.56)
**Any local adverse event**								
Overall	12.7 (8.4 to 17.1)	70.4 (57.9 to 83.0)	−2.93 (−3.70 to −2.15)	0.40	−7.41	<.001	99.03	−1.61 (−2.04 to −1.19)
Dose 1	16.2 (11.3 to 21.1)	66.7 (53.2 to 80.3)	−2.44 (−3.21 to −1.66)	0.40	−6.16	<.001	99.15	−1.34 (−1.77 to −0.92)
Dose 2	11.8 (6.6 to 17.1)	72.8 (57.4 to 88.2)	−3.15 (−3.91 to −2.39)	0.39	−8.09	<.001	98.85	−1.74 (−2.16 to −1.32)
Pain								
Overall	10.0 (6.8 to 13.1)	67.3 (53.5 to 81.2)	−3.10 (−3.86 to −2.35)	0.39	−8.02	<.001	98.78	−1.71 (−2.13 to −1.30)
Dose 1	10.0 (6.3 to 13.6)	63.3 (48.0 to 78.6)	−2.90 (−3.79 to −2.00)	0.46	−6.34	<.001	99.20	−1.60 (−2.09 to −1.10)
Dose 2	8.8 (5.2 to 12.4)	68.9 (57.0 to 80.9)	−3.21 (−3.87 to −2.56)	0.33	−9.64	<.001	98.10	−1.77 (−2.13 to −1.41)
Redness								
Overall	0.3 (0.1 to 0.5)	5.2 (2.8 to 7.5)	−2.32 (−2.80 to −1.84)	0.24	−9.53	<.001	44.18	−1.28 (−1.54 to −1.01)
Dose 1	0.3 (0.1 to 0.5)	2.2 (1.0 to 3.4)	−1.67 (−1.96 to −1.37)	0.15	−10.99	<.001	16.34	−0.92 (−1.08 to −0.76)
Dose 2	0.2 (<0.1 to 0.3)	7.2 (4.0 to 10.4)	−2.67 (−3.24 to −2.11)	0.29	−9.28	<.001	53.06	−1.47 (−1.79 to −1.16)
Swelling								
Overall	0.2 (0.1 to 0.5)	6.5 (3.5 to 9.4)	−2.59 (−3.14 to −2.05)	0.28	−9.32	<.001	55.70	−1.43 (−1.73 to −1.13)
Dose 1	0.3 (0.1 to 0.5)	3.5 (1.7 to 5.4)	−1.89 (−2.60 to −1.18)	0.36	−5.20	<.001	75.77	−1.04 (−1.43 to −0.65)
Dose 2	0.2 (0.1 to 0.4)	8.6 (4.8 to 12.3)	−2.97 (−3.54 to −2.40)	0.29	−10.25	<.001	52.38	−1.64 (−1.95 to −1.32)
Tenderness								
Overall	9.2 (4.3 to 14.0)	35.4 (14.1 to 56.7)	−1.58 (−2.09 to −1.08)	0.26	−6.14	<.001	95.23	−0.87 (−1.15 to −0.60)
Dose 1	11.2 (5.4 to 17.0)	32.6 (14.6 to 50.6)	−1.23 (−1.60 to −0.86)	0.19	−6.56	<.001	91.65	−0.68 (−0.88 to −0.47)
Dose 2	7.1 (3.0 to 11.3)	38.3 (13.2 to 63.3)	−2.08 (−2.88 to −1.27)	0.41	−5.06	<.001	97.85	−1.15 (−1.59 to −0.70)
**Any systemic adverse event**								
Overall	29.8 (23.0 to 36.5)	56.8 (47.1 to 66.5)	−1.13 (−1.61 to −0.65)	0.24	−4.62	<.001	98.40	−0.62 (−0.89 to −0.36)
Dose 1	35.2 (26.7 to 43.7)	46.3 (38.2 to 54.3)	−0.47 (−0.54 to −0.40)	0.04	−13.26	<.001	29.75	−0.26 (−0.30 to −0.22)
Dose 2	31.8 (28.7 to 35.0)	61.4 (47.4 to 75.4)	−1.36 (−1.86 to −0.86)	0.25	−5.35	<.001	98.35	−0.75 (−1.03 to −0.47)
Fever								
Overall	0.3 (0.1 to 0.5)	12.0 (1.7 to 22.3)	−2.15 (−3.10 to −1.19)	0.49	−4.42	<.001	89.52	−1.18 (−1.71 to −0.66)
Dose 1	0.4 (0.1 to 0.6)	1.3 (0.6 to 2.0)	−0.87 (−1.33 to −0.42)	0.23	−3.75	<.001	49.06	−0.48 (−0.73 to −0.23)
Dose 2	0.3 (0.1 to 0.5)	18.0 (3.7 to 32.4)	−2.54 (−3.71 to −1.37)	0.60	−4.25	<.001	92.63	−1.40 (−2.05 to −0.76)
Chills								
Overall	3.0 (1.2 to 4.9)	21.0 (13.5 to 28.6)	−1.75 (−1.82 to −1.68)	0.04	−48.46	<.001	<0.01	−0.96 (−1.00 to −0.93)
Dose 1	3.4 (1.4 to 5.4)	9.4 (6.9 to 11.8)	−0.88 (−1.33 to −0.44)	0.23	−3.89	<.001	89.39	−0.49 (−0.73 to −0.24)
Dose 2	2.6 (0.9 to 4.4)	29.3 (15.8 to 42.7)	−2.39 (−3.25 to −1.53)	0.44	−5.43	<.001	97.20	−1.32 (−1.79 to −0.84)
Fatigue								
Overall	15.9 (10.0 to 21.8)	36.1 (25.9 to 46.4)	−0.88 (−1.13 to −0.63)	0.13	−6.97	<.001	91.23	−0.49 (−0.62 to −0.35)
Dose 1	16.7 (9.8 to 23.6)	26.3 (17.9 to 34.7)	−0.45 (−0.63 to −0.28)	0.09	−4.96	<.001	82.66	−0.25 (−0.35 to −0.15)
Dose 2	14.9 (9.8 o 20.1)	43.1 (29.7 to 56.5)	−1.33 (−1.80 to −0.86)	0.24	−5.57	<.001	97.74	−0.73 (−0.99 to −0.47)
Malaise								
Overall	7.8 (4.2 to 11.4)	25.3 (13.0 to 37.5)	−1.03 (−1.75 to −0.31)	0.37	−2.81	.005	80.52	−0.57 (−0.96 to −0.17)
Dose 1	8.0 (4.2 to 11.8)	11.6 (10.2 to 13.0)	−0.22 (−0.43 to −0.01)	0.11	−2.09	.04	<0.01	−0.12 (−0.24 to −0.01)
Dose 2	6.9 (2.0 to 11.7)	34.2 (12.4 to 56.1)	−1.64 (−2.79 to −0.49)	0.59	−2.79	.005	92.55	−0.90 (−1.54 to −0.27)
Joint pain								
Overall	6.8 (4.7 to 8.9)	17.9 (13.4 to 22.3)	−1.01 (−1.25 to −0.78)	0.12	−8.41	<.001	79.14	−0.56 (−0.69 to −0.43)
Dose 1	6.6 (3.8 to 9.4)	11.4 (7.5 to 15.4)	−0.51 (−0.65 to −0.37)	0.07	−7.04	<.001	45.37	−0.28 (−0.36 to −0.20)
Dose 2	6.3 (4.4 to 8.2)	23.3 (15.5 to 31.0)	−1.41 (−1.89 to −0.94)	0.24	−5.80	<.001	95.27	−0.78 (−1.04 to −0.52)
Muscle pain								
Overall	8.2 (5.5 to 11.0)	28.5 (21.2 to 35.9)	−1.30 (−1.56 to −1.05)	0.13	−9.96	<.001	86.17	−0.72 (−0.86 to −0.58)
Dose 1	9.1 (6.0 to 12.1)	19.1 (13.8 to 24.4)	−0.71 (−0.84 to −0.59)	0.07	−10.88	<.001	49.54	−0.39 (−0.46 to −0.33)
Dose 2	7.2 (4.7 to 9.7)	37.0 (24.5 to 49.4)	−1.89 (−2.40 to −1.37)	0.26	−7.19	<.001	96.84	−1.04 (−1.32 to −0.76)
Headache								
Overall	18.4 (14.5 to 22.4)	36.5 (30.6 to 42.5)	−0.83 (−1.16 to −0.51)	0.17	−5.00	<.001	95.53	−0.46 (−0.64 to −0.28)
Dose 1	19.3 (13.6 to 25.1)	28.5 (24.3 to 32.6)	−0.32 (−0.44 to −0.19)	0.07	−4.86	<.001	65.02	−0.17 (−0.24 to −0.10)
Dose 2	16.2 (12.5 to 19.8)	43.0 (31.7 to 54.2)	−1.33 (−1.91 to −0.76)	0.29	−4.53	<.001	98.64	−0.74 (−1.05 to −0.42)
Nausea and/or vomiting								
Overall	2.9 (1.0 to 4.9)	6.2 (2.9 to 9.6)	−0.63 (−0.85 to −0.40)	0.12	−5.42	<.001	37.32	−0.35 (−0.47 to −0.22)
Dose 1	3.1 (1.0 to 5.2)	4.2 (1.9 to 6.4)	0.19 (−0.34 to 0.72)	0.27	0.71	.48	82.14	0.11 (−0.19 to 0.40)
Dose 2	2.8 (0.8 to 4.7)	7.7 (2.7 to 12.8)	−0.96 (−1.60 to −0.33)	0.32	−2.99	.003	89.95	−0.53 (−0.88 to −0.18)
Diarrhea								
Overall	4.3 (0.5 to 8.1)	4.3 (<0.1 to 9.0)	−0.15 (−0.30 to 0.01)	0.08	−1.87	.06	<0.01	−0.08 (−0.17 to 0.01)
Dose 1	3.1 (<0.1 to 7.8)	3.5 (<0.1 to 8.4)	−0.04 (−0.19 to 0.11)	0.08	−0.55	.58	<0.01	−0.02 (−0.10 to 0.06)
Dose 2	3.3 (<0.1 to 7.0)	3.8 (<0.1 to 8.8)	−0.28 (−0.44 to −0.11)	0.08	−3.30	<.001	<0.01	−0.15 (−0.24 to −0.06)

^a^ No statistical tests for the presence of adverse events were done because for proportions, the null hypothesis (ie, that there were no cases in the population) could be rejected whenever a single case was reported.

^b^ Although the statistical tests of the pooled logarithmic odds ratios included multiple comparisons, adjusting for this multiplicity by Bonferroni correction would tend to make significant group differences not significant and therefore strengthen the conclusion that a substantial fraction of the adverse events experienced after vaccination were actually nocebo responses. Thus, the more conservative analytical strategy in this case was to not adjust for multiple comparisons.

After the second dose, however, the random-effects pooled log ORs and equivalent standardized mean differences indicated larger differences between the vaccine and placebo groups in reporting of AEs (OR, −1.36; 95% CI, −1.86 to −0.86; *P* < .001). Except for nausea and diarrhea, effects were large or very large for all AE categories (Table 3), with a standardized mean difference of −0.75 (95% CI, 1.03 to −0.47) for any systemic AE and −1.74 (95% CI, −2.16 to −1.32) for any local AE. Heterogeneity of the included studies was very high (*I*^2^≥50%) for most AE categories.

The results of the equivalent log OR meta-analysis controlling for methodologic quality aspects are provided in the eTable 3 in the Supplement. There were no major differences in the results when controlling for risk of bias, and heterogeneity remained high.

### Severity of AEs

Aside from the presence or absence of AEs, the severity of AEs could serve as an additional indicator to quantify the influence of nocebo responses. In exploratory analyses of the 2 largest trials reporting details on severity,^38,39^ we found that the proportion of severity grades for participants reporting any systemic AEs after the first dose were similar in the placebo and vaccine groups (eFigures 1 and 2 in the Supplement). However, whereas the pattern of severity grading after the second dose stayed the same for placebo participants, there were proportionally more moderate and severe AEs in the vaccine groups after the second dose.

## Discussion

This systematic review and meta-analysis evaluated the frequency of solicited AEs in the placebo groups of randomized clinical trials investigating COVID-19 vaccines. The 12 analyzed trials^38,39,40,41,42,43,44,45,46,47,48,49^ included different types of vaccines (mRNA, viral vector, or protein-based) and different clinical trial phases. We found that 76.0% of systemic AEs and 24.3% of local AEs after the first vaccination could be attributed to nocebo responses. After the second vaccination, 51.8% of systemic AEs and 16.2% of local AEs were attributable to nocebo responses. Headache and fatigue were the most common AEs in the placebo groups, experienced by 19.3% and 16.7% of participants, respectively, after the first dose.

Of interest, AE frequencies in the placebo groups were lower after the second dose than after the first dose, although the opposite was true for the vaccine groups. We hypothesize that (1) the second dose of the vaccines may have produced both a more robust immune response and a correspondingly more robust set of AEs and that (2) participants in the vaccine arms, after experiencing more AEs after the first dose than did participants in the control groups, had higher expectations for AEs after the second dose compared with participants in the placebo arms.^51^

The finding of decreased AEs in placebo recipients but increased AEs in vaccine recipients after the second dose was supported by our evaluation of standardized mean differences. Although we found significantly higher AE rates in the vaccine groups for nearly every evaluated symptom category, group differences were particularly large after the second dose. After the first dose, however, the group differences for most systemic AEs were small. Headache, fatigue, malaise, and joint pain were common in both groups and seem to have been particularly associated with nocebo. Furthermore, exploratory analyses suggested that nocebo responses may produce AEs of severity grades similar to those of active vaccines after the first dose.

### Healthcare Implications

Given the large number of people who have received or will receive a COVID-19 vaccine, this study’s findings are important for the general population worldwide. Common nonspecific symptoms such as headache and fatigue, which the study’s findings showed to be particularly associated with nocebo, are listed among the most common AEs after COVID-19 vaccination in many information leaflets.^52,53,54^ There is evidence that this sort of information may increase nocebo mechanisms such as AE-related anxiety and expectations.^17^ Furthermore, the information might cause a misattribution of commonly experienced nonspecific symptoms (eg, headache or fatigue^55^) as specific AEs due to vaccination, even if these symptoms might have occurred in the absence of receiving any treatment.^56^ Thus, the current way of informing the public about potential vaccine AEs via leaflets and in the media may prompt or further increase nocebo responses.^30^ Nonetheless, it is ethically necessary to fully inform participants about the vaccines’ potential AEs. Emergent data suggest that full disclosure and education about nocebo responses may be helpful.^19,20^ For example, adding simple but accurate information about nocebo responses to the usual informed consent procedure (eg, “participants in the placebo arm of the randomized clinical trials testing this intervention reported similar AEs, probably because of worry and anxiety”) helped reduce medication-related AEs in a clinical population.^20^ Highlighting the probability of not experiencing AEs might also be beneficial.^21^ Although more research on these communication strategies is needed, such honest information adds to full disclosure and is unlikely to cause harm. In addition, informing the public about the potential for nocebo responses may help reduce worries about COVID-19 vaccination, which might decrease vaccination hesitancy.^9,31^

### Limitations

This study has limitations. The relatively small number of included trials and their high heterogeneity must be considered when interpreting the results. The high heterogeneity may have been caused by (1) different AE assessment methods (ie, the trials used different symptom checklists and did not assess the attributability of symptoms in a standardized way), (2) different types of tested vaccine (mRNA, viral vector, or protein-based), or (3) different probabilities of receiving a vaccine vs a placebo in the different trials (ie, probabilities to receive a placebo ranged from 14% to 50%). Standardization of AE assessment could alleviate the first of these potential causes,^57^ and larger meta-analyses should investigate the role of the second and third causes using meta-regression. However, because our analyses included randomized clinical trials of different clinical trial phases and different vaccine manufacturing types that are currently used in practice, we believe the findings of high nocebo responses are relevant for COVID-19 vaccination in everyday health care.

## Conclusions

In this systematic review and meta-analysis, approximately one-third of placebo recipients in COVID-19 vaccine randomized clinical trials reported at least 1 systemic AE after both the first and the second dose, with headache and fatigue being the most common. This nocebo response accounted for 76.0% of systemic AEs after the first dose of COVID-19 vaccine, and for 51.8% after the second dose. Public vaccination programs should consider these high nocebo responses.

## Notes

Supplement. **eAppendix.** Search Strategy **eTable 1.** Adverse Events in Placebo and Vaccine Groups of the Analyzed Trials **eTable 2.** Mixed Meta-analysis of Proportions **eTable 3.** Mixed Meta-analysis of Logarithmized Odds Ratios **eFigure 1.** Adverse Event Severity Grading in the Phase-3 Trial of the Moderna Vaccine **eFigure 2.** Adverse Event Severity Grading in the Phase-3 Trial of the Novavax Vaccine
eReferences
