# Error in Figure 1

**DOI:** 10.1001/jamanetworkopen.2022.1277

**Published:** 2022-02-11

In the Original Investigation titled “Frequency of Adverse Events in the Placebo Arms of COVID-19 Vaccine Trials: A Systematic Review and Meta-Analysis,”^1^ published January 18, 2022, the following 2 boxes in Figure 1 were incorrectly transposed: 87 articles were screened, and 27 articles were sought for retrieval. This article has been corrected.^1^

## References

[1] Haas JW, Bender FL, Ballou S, . Frequency of adverse events in the placebo arms of COVID-19 vaccine trials: a systematic review and meta-analysis. JAMA Netw Open. 2022;5(1):e2143955. doi:10.1001/jamanetworkopen.2021.43955 35040967 PMC8767431

