# Supplementary material for: Frequency of Adverse Events in the Placebo Arms of COVID-19 Vaccine Trials: A Systematic Review and Meta-analysis
Source: JAMA Netw Open. 2022 Jan 18;5(1):e2143955. doi: 10.1001/jamanetworkopen.2021.43955 (PMC8767431; doi:10.1001/jamanetworkopen.2021.43955)
Supplement: Supplement. — eAppendix. Search Strategy eTable 1. Adverse Events in Placebo and Vaccine Groups of the Analyzed Trials eTable 2. Mixed Meta-analysis of Proportions eTable 3. Mixed Meta-analysis of Logarithmized Odds Ratios eFigure 1. Adverse Event Severity Grading in the Phase-3 Trial of the Moderna Vaccine eFigure 2. Adverse Event Severity Grading in the Phase-3 Trial of the Novavax Vaccine eReferences [file jamanetwopen-e2143955-s001.pdf]

Supplementary Online Content

Haas JW, Bender FL, Ballou S, et al. Frequency of adverse events in the placebo arms of COVID-19 vaccine trials: a systematic review and meta-analysis. *JAMA Netw Open*. 2022;5(1):e2143955. doi:10.1001/jamanetworkopen.2021.43955

eAppendix. Search Strategy

eTable 1. Adverse Events in Placebo and Vaccine Groups of the Analyzed Trials

eTable 2. Mixed Meta-analysis of Proportions

eTable 3. Mixed Meta-analysis of Logarithmized Odds Ratios

eFigure 1. Adverse Event Severity Grading in the Phase-3 Trial of the Moderna Vaccine

eFigure 2. Adverse Event Severity Grading in the Phase-3 Trial of the Novavax Vaccine

eReferences

This supplementary material has been provided by the authors to give readers additional information about their work.

© 2022 Haas JW et al. *JAMA Network Open*.

eAppendix. Search Strategy

A systematic literature search of studies published up to July 14, 2021 was conducted across the Medline database (PubMed) and the Cochrane Central Register of Controlled Trials (CENTRAL). As a first step, these databases were searched for Medical Sub Heading (Mesh) terms ("COVID-19 Vaccines" [Mesh] AND "Randomized Controlled Trial" [Publication Type]). In addition, a free text search was conducted using the following keywords:

> (vaccination[Title/Abstract] OR inoculation[Title/Abstract] OR immunis*[Title/Abstract] OR vaccine[Title/Abstract] OR immuniz*[Title/Abstract]) AND (COVID-19[Title/Abstract] OR COVID[Title/Abstract] OR sars[Title/Abstract] OR sars-cov-2*[Title/Abstract]) AND (placebo*[Title/Abstract] OR saline[Title/Abstract]) AND (adverse events[Title/Abstract] OR AE[Title/Abstract] OR AEs[Title/Abstract] OR AEFI[Title/Abstract] OR safety[Title/Abstract] OR tolera*[Title/Abstract] OR side-effects[Title/Abstract] OR adverse reactions[Title/Abstract])

© 2022 Haas JW et al. *JAMA Network Open*.

Supplemental Data

eTable 1

Numbers and percentages of solicited adverse events in the twelve analyzed randomized controlled trials of COVID-19 vaccines

	N with AE reports	Any AEs, n (%)	Local adverse events, n (%)					Systemic adverse events, n (%)									
			Any	Pain	Redness	Swelling	Tender-ness	Any	Fever	Chills	Fatigue	Malaise	Joint pain	Muscle pain	Headache	Nausea / vomiting	Diarrhea
**mRNA vaccines**																	
Baden et al., 2021																	
1st dose P	15155	7284 (48)	2997 (20)	2658 (18)	67 (0.4)	52 (0.3)	722 (5)	6399 (42)	44 (0.3)	878 (6)	4133 (27)	NA	1783 (12)	2071 (14)	4027 (27)	1074 (7)	NA
1st dose V	15168	13319 (88)	12765 (84)	12690 (84)	430 (3)	932 (6)	1553 (10)	8320 (55)	115 (1)	1253 (8)	5635 (37)	NA	2511 (17)	3441 (23)	4951 (33)	1262 (8)	NA
2nd dose P	14566	6232 (43)	2735 (19)	2477 (17)	56 (0.4)	49 (0.3)	567 (4)	5323 (37)	43 (0.3)	809 (6)	3403 (23)	NA	1569 (11)	1809 (12)	3410 (23)	934 (6)	NA
2nd dose V	14677	13534 (92)	13006 (89)	12943 (88)	1257 (9)	1789 (12)	2090 (14)	11652 (79)	2278 (16)	6482 (44)	9582 (65)	NA	6284 (43)	8508 (58)	8602 (59)	2785 (19)	NA
Chu et al., 2021																	
1st dose P	199	77 (39)	27 (14)	21 (11)	1 (0.5)	1 (0.5)	5 (3)	64 (32)	0 (0)	6 (3)	35 (18)	NA	10 (5)	14 (7)	36 (18)	10 (5)	NA
1st dose V	200	176 (88)	168 (84)	166 (83)	0 (0)	8 (4)	18 (9)	89 (45)	1 (0.5)	10 (5)	50 (25)	NA	18 (9)	28 (14)	43 (22)	6 (3)	NA
2nd dose P	193	66 (34)	16 (8)	15 (8)	5 (3)	1 (0.5)	1 (0.5)	58 (30)	1 (0.5)	5 (3)	41 (21)	NA	13 (7)	15 (8)	33 (17)	2 (1)	NA
2nd dose V	199	178 (89)	170 (85)	169 (85)	15 (8)	21 (11)	20 (10)	153 (77)	26 (13)	78 (39)	128 (64)	NA	77 (39)	104 (52)	104 (52)	41 (21)	NA
Polack et al., 2020																	
1st dose P	4040-4090	NA	525 (13)	488 (12)	45 (1)	32 (1)	NA	1922 (47)	27 (1)	203 (5)	1172 (29)	NA	247 (6)	398 (10)	1100 (27)	37 (1)	388 (10)
1st dose V	4040-4093	NA	3216 (79)	3186 (79)	189 (5)	250 (6)	NA	2421 (59)	111 (3)	434 (11)	1700 (42)	NA	406 (10)	738 (18)	1413 (35)	37 (1)	402 (10)
2nd dose P	3699-3749	NA	396 (11)	372 (10)	26 (1)	16 (0.4)	NA	1267 (34)	14 (0.4)	125 (3)	756 (20)	NA	170 (5)	260 (7)	735 (20)	30 (1)	276 (7)
2nd dose V	3705-3758	NA	2748 (73)	2730 (74)	243 (7)	256 (7)	NA	2627 (70)	512 (14)	1114 (30)	2086 (56)	NA	772 (21)	1260 (34)	1732 (47)	51 (1)	356 (10)
Li et al., 2021																	
Overall P	48	6 (13)	2 (4)	2 (4)	0 (0)	0 (0)	NA	5 (10)	0 (0)	0 (0)	0 (0)	1 (2)	1 (2)	0 (0)	3 (6)	0 (0)	1 (2)
Overall V	48	46 (95)	45 (94)	44 (92)	12 (25)	12 (25)	NA	39 (81)	34 (71)	11 (23)	24 (50)	13 (27)	11 (23)	11 (23)	21 (44)	3 (6)	1 (2)
1st dose P	48	NA	NA	1 (2)	0 (0)	0 (0)	NA	NA	0 (0)	0 (0)	0 (0)	1 (2)	0 (0)	0 (0)	1 (2)	0 (0)	0 (0)
1st dose V	48	NA	NA	41 (85)	10 (21)	5 (10)	NA	NA	19 (40)	6 (13)	11 (23)	7 (15)	5 (10)	7 (15)	10 (21)	3 (6)	0 (0)
2nd dose P	48	NA	NA	1 (2)	0 (0)	0 (0)	NA	NA	0 (0)	0 (0)	0 (0)	0 (0)	1 (2)	0 (0)	2 (4)	0 (0)	1 (2)
2nd dose V	47	NA	NA	35 (74)	5 (11)	9 (19)	NA	NA	39 (83)	8 (17)	22 (47)	10 (21)	11 (23)	8 (17)	17 (36)	1 (2)	0 (0)
Walsh et al., 2020																	
Overall P	39	10 (26)	NA	NA	NA	NA	NA	NA	NA	NA	NA	NA	NA	NA	NA	NA	NA
Overall V	48	17 (35)	NA	NA	NA	NA	NA	NA	NA	NA	NA	NA	NA	NA	NA	NA	NA
1st dose P	36-39	NA	NA	1 (3)	0 (0)	0 (0)	NA	NA	0 (0)	2 (6)	11 (31)	NA	2 (6)	3 (8)	5 (14)	0 (0)	1 (3)
1st dose V	48	NA	NA	43 (90)	3 (6)	4 (8)	NA	NA	6 (13)	13 (27)	20 (42)	NA	3 (6)	11 (23)	18 (38)	1 (2)	2 (4)
2nd dose P	36	NA	NA	5 (14)	0 (0)	0 (0)	NA	NA	0 (0)	1 (3)	10 (28)	NA	1 (3)	1 (3)	3 (8)	1 (3)	0 (0)
2nd dose V	48	NA	NA	39 (81)	3 (6)	6 (13)	NA	NA	16 (33)	21 (44)	32 (67)	NA	8 (17)	21 (44)	32 (67)	1 (2)	3 (6)
**Viral vector vaccines**																	
Madhi et al., 2021																	
1st dose P	935-968	NA	NA	NA	0 (0)	0 (0)	122 (13)	NA	5 (1)	25 (3)	181 (19)	NA	115 (12)	137 (14)	250 (26)	NA	NA
1st dose V	918-952	NA	NA	NA	1 (0.1)	2 (0.2)	345 (36)	NA	5 (1)	87 (9)	304 (32)	NA	215 (23)	290 (30)	361 (38)	NA	NA
2nd dose P	886-941	NA	NA	NA	0 (0)	0 (0)	75 (8)	NA	3 (0.3)	14 (2)	114 (12)	NA	70 (7)	79 (8)	161 (17)	NA	NA
2nd dose V	877-926	NA	NA	NA	1 (0.1)	1 (0.1)	245 (27)	NA	1 (0.1)	26 (3)	158 (17)	NA	95 (10)	153 (17)	190 (21)	NA	NA
Sadoff et al., 2021																	
Placebo	163	48 (29)	19 (12)	NA	NA	NA	NA	40 (25)	0 (0)	NA	NA	NA	NA	NA	NA	NA	NA
Vaccine	323	220 (68)	170 (53)	NA	NA	NA	NA	179 (55)	32 (10)	NA	NA	NA	NA	NA	NA	NA	NA
**Protein-based vaccines**																	
Goepfert et al., 2021																	
1st dose P	29	NA	NA	4 (14)	0 (0)	0 (0)	NA	NA	0 (0)	NA	NA	5 (17)	NA	2 (7)	7 (24)	NA	NA
1st dose V	80	NA	NA	53 (67)	1 (1)	1 (1)	NA	NA	1 (1)	NA	NA	16 (20)	NA	22 (28)	20 (25)	NA	NA
2nd dose P	29	NA	NA	3 (10)	1 (3)	0 (0)	NA	NA	0 (0)	NA	NA	3 (10)	NA	3 (10)	3 (10)	NA	NA
2nd dose V	80	NA	NA	70 (89)	19 (24)	15 (19)	NA	NA	28 (36)	NA	NA	59 (75)	NA	60 (75)	57 (71)	NA	NA
Heath et al., 2021																	
1st dose P	1350	NA	2828 (21)	138 (10)	5 (0.4)	7 (1)	236 (17)	512 (38)	20 (1)	NA	259 (19)	130 (10)	66 (5)	192 (14)	290 (21)	73 (5)	NA
1st dose V	1364	NA	809 (59)	419 (31)	26 (2)	12 (1)	749 (55)	649 (48)	31 (2)	NA	280 (21)	158 (12)	90 (7)	304 (22)	334 (24)	71 (5)	NA
2nd dose P	1335	NA	227 (17)	122 (9)	3 (0.2)	4 (0.3)	187 (14)	411 (31)	11 (1)	NA	223 (17)	123 (9)	68 (5)	130 (10)	239 (18)	51 (4)	NA

© 2022 Haas JW et al. *JAMA Network Open*.

2nd dose V	1348	NA	1081 (80)	700 (52)	112 (8)	100 (7)	1033 (77)	871 (65)	69 (5)	NA	553 (41)	425 (32)	231 (17)	554 (41)	549 (41)	144 (11)	NA
Keech et al., 2020																	
1st dose P	23	NA	7 (30)	3 (13)	0 (0)	NA	7 (30)	9 (39)	0 (0)	NA	4 (17)	2 (9)	1 (4)	2 (9)	7 (30)	1 (4)	NA
1st dose V	26	NA	18 (70)	10 (38)	0 (0)	NA	17 (65)	12 (46)	0 (0)	NA	8 (31)	3 (12)	1 (4)	6 (23)	6 (23)	1 (4)	NA
2nd dose P	21	NA	4 (19)	2 (10)	1 (5)	NA	2 (10)	7 (33)	0 (0)	NA	3 (14)	3 (14)	2 (10)	3 (14)	6 (29)	0 (0)	NA
2nd dose V	26	NA	24 (92)	15 (58)	2 (8)	NA	21 (81)	17 (65)	0 (0)	NA	12 (46)	9 (35)	7 (27)	12 (46)	12 (46)	2 (8)	NA
Richmond et al., 2021																	
1st dose P	30	NA	1 (3)	1 (3)	0 (0)	0 (0)	NA	3 (10)	0 (0)	NA	0 (0)	NA	NA	1 (3)	1 (3)	0 (0)	0 (0)
1st dose V	16	NA	7 (44)	5 (31)	1 (6)	1 (6)	NA	3 (19)	0 (0)	NA	0 (0)	NA	NA	0 (0)	3 (19)	0 (0)	0 (0)
2nd dose P	30	NA	0 (0)	0 (0)	0 (0)	0 (0)	NA	6 (20)	0 (0)	NA	2 (7)	NA	NA	1 (3)	3 (10)	0 (0)	1 (3)
2nd dose V	16	NA	7 (44)	7 (44)	2 (13)	1 (6)	NA	5 (31)	0 (0)	NA	2 (13)	NA	NA	1 (6)	2 (13)	0 (0)	0 (0)
Shinde et al., 2021																	
1st dose P	484	NA	96 (20)	73 (15)	2 (0.4)	2 (0.4)	60 (12)	167 (35)	7 (1)	NA	59 (12)	44 (9)	41 (8)	49 (19)	114 (24)	31 (6)	NA
1st dose V	484	NA	197 (41)	181 (37)	7 (1)	10 (2)	119 (25)	188 (39)	9 (2)	NA	75 (15)	52 (11)	67 (14)	89 (18)	118 (24)	41 (8)	NA
2nd dose P	470	NA	64 (14)	53 (11)	0 (0)	2 (0.4)	41 (9)	131 (28)	7 (1)	NA	53 (11)	38 (8)	34 (7)	38 (8)	89 (19)	36 (8)	NA
2nd dose V	471	NA	185 (39)	172 (37)	6 (1)	10 (2)	112 (24)	162 (34)	17 (4)	NA	68 (14)	46 (10)	65 (14)	82 (17)	97 (21)	32 (7)	NA

Note: AE = adverse event; P = placebo; V = vaccine. Decimals are provided for percentages ≤ 0.5.

© 2022 Haas JW et al. *JAMA Network Open*.

Supplemental Results

eTable 2

Coefficients of the mixed random-effects meta-analysis of adverse event proportions in the placebo groups, controlling for risk of bias

	k	Proportion	SE	95% CI Lower	95% CI Upper	I^2, %
**Any Adverse Event**	5	0.218	0.11	0.003	0.432	89.48
**Any Local AE**	9	0.110	0.05	0.012	0.208	98.02
Dose 1	8	0.182	0.02	0.140	0.225	97.00
Dose 2	8	0.150	0.02	0.108	0.192	97.29
**Pain**	10	0.042	0.04	−0.044	0.127	94.05
Dose 1	10	0.137	0.02	0.105	0.170	93.94
Dose 2	10	0.118	0.02	0.079	0.157	96.33
**Redness**	11	< 0.001	< 0.01	−0.007	0.007	91.61
Dose 1	11	0.004	< 0.01	< 0.001	0.007	94.51
Dose 2	11	0.001	< 0.01	< 0.001	0.002	63.56
**Swelling**	10	< 0.001	< 0.01	−0.007	0.007	91.26
Dose 1	10	0.004	< 0.01	< 0.001	0.006	92.84
Dose 2	10	0.003	< 0.01	< 0.001	0.005	91.13
**Tenderness**	6	0.102	0.02	0.055	0.149	97.24
Dose 1	6	0.117	0.03	0.065	0.170	97.57
Dose 2	6	0.086	0.02	0.044	0.127	96.98
**Any Systemic AE**	9	0.204	0.07	0.068	0.340	97.89
Dose 1	8	0.405	0.04	0.329	0.482	98.29
Dose 2	8	0.326	0.02	0.290	0.362	92.27
**Fever**	12	0.002	< 0.01	−0.003	0.007	63.81
Dose 1	12	0.006	< 0.01	0.003	0.010	77.57
Dose 2	12	0.004	< 0.01	0.002	0.006	52.61
**Chills**	6	< 0.001	0.02	−0.033	0.033	95.16
Dose 1	6	0.045	0.01	0.027	0.063	93.66
Dose 2	6	0.035	0.01	0.014	0.056	96.41
**Fatigue**	10	< 0.001	0.07	−0.146	0.146	98.36
Dose 1	10	0.213	0.04	0.131	0.295	98.93
Dose 2	10	0.169	0.03	0.117	0.221	97.60
**Malaise**	5	0.021	0.02	−0.020	0.061	0.00[1]
Dose 1	5	0.096	0.01	0.082	0.109	0.17[2]
Dose 2	5	0.089	0.01	0.076	0.102	0.00[3]
**Joint pain**	9	0.021	0.03	−0.044	0.085	95.68
Dose 1	9	0.086	0.01	0.059	0.113	96.51
Dose 2	9	0.070	0.01	0.048	0.093	95.48
**Muscle pain**	11	< 0.001	0.02	−0.035	0.035	83.95
Dose 1	11	0.122	0.01	0.104	0.141	85.14
Dose 2	11	0.092	0.01	0.073	0.112	88.88
**Headache**	11	0.063	0.04	−0.019	0.144	81.00

© 2022 Haas JW et al. *JAMA Network Open*.

	k	Proportion	SE	95% CI Lower	Upper	I², %
Dose 1	11	0.250	0.02	0.219	0.281	90.41
Dose 2	11	0.192	0.01	0.168	0.217	86.82
**Nausea/vomiting**	9	< 0.001	0.02	−0.048	0.048	97.79
Dose 1	9	0.049	0.01	0.022	0.076	98.53
Dose 2	9	0.045	0.01	0.020	0.071	98.05
**Diarrhea**	4	0.021	0.02	−0.020	0.061	0.00[4]
Dose 1	4	0.096	0.01	0.087	0.105	0.00[5]
Dose 2	4	0.075	0.01	0.066	0.084	0.50[5]

Note. AE = adverse event; k = number of studies included in analyses; SE = standard error; CI = confidence interval. Analyses control for risk of bias due to selective reporting (no AE reports over both doses or time interval of AE assessment > 7 days), incomplete outcome data (high drop-out rate in placebo group) and other source of bias (inclusion of sentinel participants in AE reports). [1]95 % CI 0.00–99.23; [2]95 % CI 0.00–99.71; [3]95 % CI 0.00–94.33; [4]95 % CI 0.00–98.97; [5]95 % CI 0.00–99.90.

eTable 3

Coefficients of the mixed random-effects meta-analysis of logarithmic odds ratios to compare the frequency of adverse events in the placebo and vaccine groups, controlling for risk of bias

	Log OR	SE	z	p	95 % CI Lower	Upper	I², %	d
**Any Adverse Event**	−4.16	1.33	−3.12	.002	−6.78	−1.55	91.83	−2.30
**Any Local AE**	−3.73	1.29	−2.89	.004	−6.25	−1.20	99.62	−2.05
Dose 1	−2.26	0.49	−4.63	< .001	−3.22	−1.30	99.41	−1.25
Dose 2	−2.77	0.43	−6.42	< .001	−3.62	−1.93	99.08	−1.53
**Pain**	−5.53	1.22	−4.55	< .001	−7.92	−3.15	98.54	−3.05
Dose 1	−2.32	0.46	−5.02	< .001	−3.22	−1.41	99.05	−1.28
Dose 2	−2.89	0.41	−7.13	< .001	−3.68	−2.09	98.56	−1.59
**Redness**	−3.50	1.52	−2.31	.02	−6.48	−0.53	54.33	−1.93
Dose 1	−1.66	0.16	−10.35	< .001	−1.97	−1.34	22.75[1]	−0.91
Dose 2	−2.81	0.31	−9.09	< .001	−3.42	−2.20	58.46	−1.55
**Swelling**	−3.50	1.56	−2.25	.02	−6.56	−0.45	63.99	−1.93
Dose 1	−1.79	0.44	−4.09	< .001	−2.65	−0.94	82.31	−0.99
Dose 2	−2.99	0.33	−9.03	< .001	−3.64	−2.34	60.32	−1.65
**Tenderness**	−1.46	0.30	−4.82	< .001	−2.06	−0.87	97.39	−0.81
Dose 1	−1.20	0.23	−5.29	< .001	−1.64	−0.76	95.74	−0.66
Dose 2	−1.76	0.42	−4.18	< .001	−2.59	−0.94	98.44	−0.97
**Any Systemic AE**	−2.43	0.63	−3.87	< .001	−3.66	−1.20	98.80	−1.34
Dose 1	−0.46	0.04	−10.83	< .001	−0.54	−0.37	50.96	−0.25
Dose 2	−1.29	0.35	−3.67	< .001	−1.98	−0.60	99.14	−0.71
**Fever**	−4.35	1.48	−2.95	.003	−7.24	−1.46	91.00	−2.40

© 2022 Haas JW et al. *JAMA Network Open*.

	Log OR	SE	z	p	95 % CI Lower	95 % CI Upper	I², %	d
Dose 1	−0.76	0.24	−3.14	.002	−1.24	−0.29	56.79	−0.42
Dose 2	−2.29	0.72	−3.21	.001	−3.69	−0.89	94.06	−1.26
**Chills**	−3.39	1.46	−2.32	.02	−6.26	−0.53	0.00[2]	−1.87
Dose 1	−0.81	0.28	−2.96	.003	−1.35	−0.28	94.22	−0.45
Dose 2	−1.97	0.54	−3.68	< .001	−3.02	−0.92	98.18	−1.09
**Fatigue**	−4.58	1.48	−3.09	.002	−7.48	−1.68	93.58	−2.52
Dose 1	−0.43	0.11	−4.15	< .001	−0.64	−0.23	88.86	−0.24
Dose 2	−1.09	0.28	−3.86	< .001	−1.65	−0.54	98.32	−0.60
**Malaise**	−2.86	1.22	−2.35	.02	−5.25	−0.47	86.85	−1.58
Dose 1	−0.20	0.11	−1.89	.06	−0.41	0.01	0.00[3]	−0.11
Dose 2	−1.56	0.84	−1.86	.06	−3.21	0.08	97.13	−0.86
**Joint pain**	−2.64	1.09	−2.41	.02	−4.78	−0.49	83.84	−1.45
Dose 1	−0.51	0.08	−6.73	< .001	−0.65	−0.36	57.77	−0.28
Dose 2	−1.21	0.27	−4.44	< .001	−1.74	−0.68	96.62	−0.67
**Muscle pain**	−3.39	1.49	−2.29	.02	−6.30	−0.48	86.90	−1.87
Dose 1	−0.70	0.07	−10.37	< .001	−0.84	−0.57	58.31	−0.39
Dose 2	−1.73	0.31	−5.54	< .001	−2.34	−1.12	97.74	−0.95
**Headache**	−2.46	0.74	−3.32	< .001	< .001	−3.91	94.09	−1.35
Dose 1	−0.30	0.08	−4.00	< .001	−0.44	−0.15	76.93	−0.16
Dose 2	−1.12	0.38	−2.96	.003	−1.86	−0.38	99.08	−0.62
**Nausea/vomiting**	−2.01	1.54	−1.31	.19	−5.02	1.00	48.75[4]	−1.11
Dose 1	0.29	0.36	0.82	.41	−0.41	1.00	90.65	0.16
Dose 2	−0.77	0.32	−2.42	.02	−1.40	−0.15	91.00	−0.43
**Diarrhea**	< −0.01	1.43	< −0.01	1.00	−2.80	2.80	0.00[5]	0.00
Dose 1	−0.04	0.08	−0.53	.60	−0.19	0.11	0.00[6]	−0.02
Dose 2	−0.28	0.08	−3.30	< .001	−0.44	−0.11	0.00[7]	−0.15

Note. AE = adverse event; log OR = logarithmic odds ratio; SE = standard error; CI = confidence interval; d = standard mean difference. Analyses control for risk of bias due to selective reporting (no AE reports over both doses or time interval of AE assessment > 7 days), incomplete outcome data (high drop-out rate in placebo group) and other source of bias (inclusion of sentinel participants in AE reports). [1]95 % CI 0.00–49.22; [2]95 % CI 0.00–98.98; [3]95 % CI 0.00–0.00; [4]95 % CI 0.00–94.99; [5]95 % CI 0.00–97.30; [6]95 % CI 0.00–85.35; [7]95 % CI 0.00–97.52.

© 2022 Haas JW et al. *JAMA Network Open*.

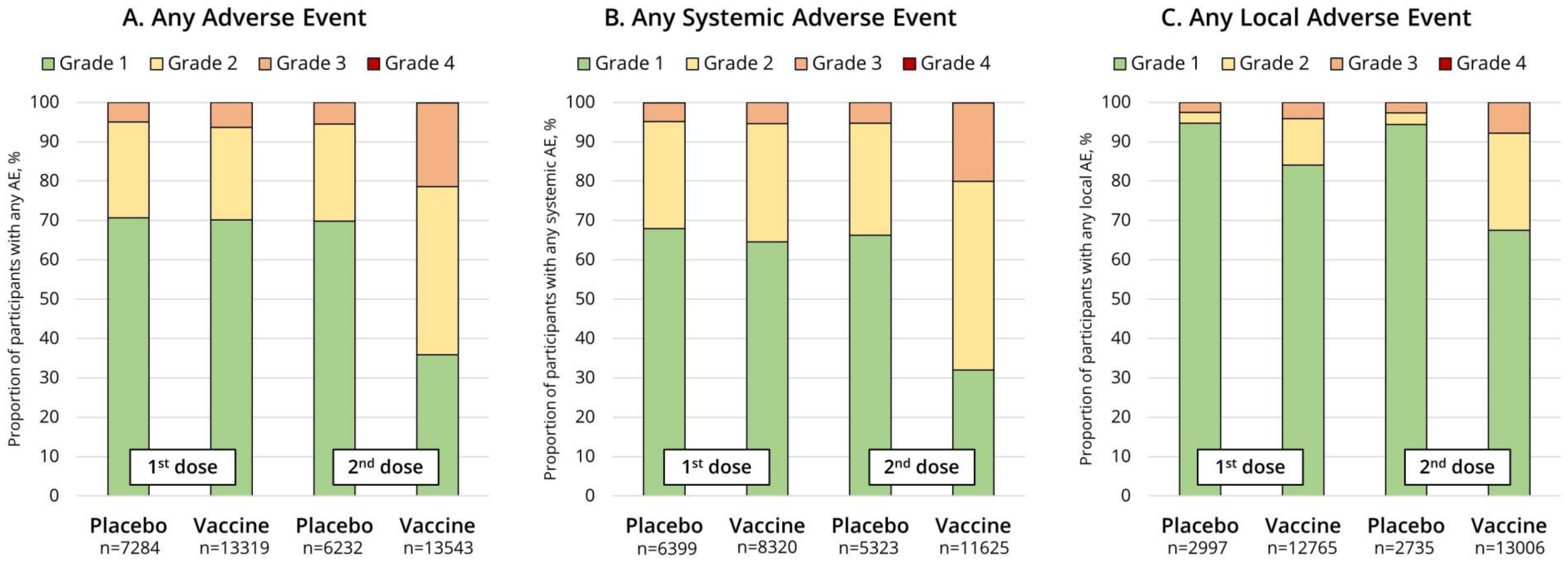

eFigure 1. Adverse event severity grading in the phase-3 trial of the Moderna vaccine (Baden et al., 2021). Plots show fractions of severity grades within participants who reported any adverse events (A), any systemic adverse event (B) or any local adverse event (C). Grade 1 = mild, grade 2 = moderate, grade 3 = severe, grade 4 = life-threatening.

© 2022 Haas JW et al. *JAMA Network Open*.

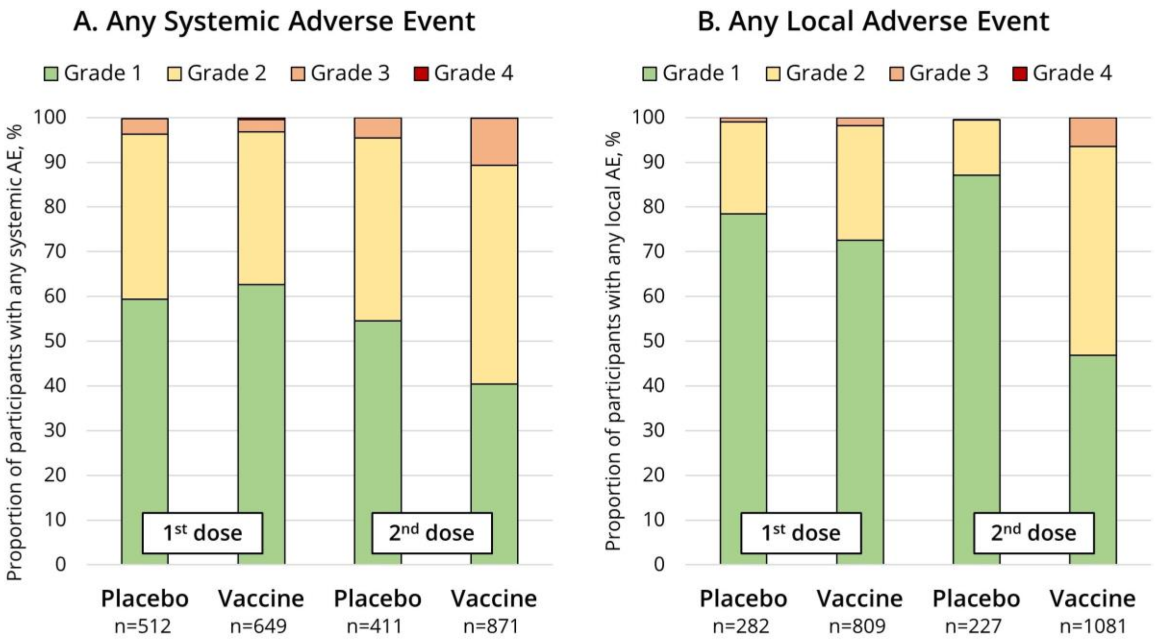

eFigure 2. Adverse event severity grading in the phase-3 trial of the Novavax vaccine (Heath et al., 2021). Plots show fractions of severity grades within participants who reported any adverse events (A), any systemic adverse event (B) or any local adverse event (C). Grade 1 = mild, grade 2 = moderate, grade 3 = severe, grade 4 = life-threatening.

© 2022 Haas JW et al. *JAMA Network Open*.

eReferences

Baden, L. R., El Sahly, H. M., Essink, B., Kotloff, K., Frey, S., Novak, R., Diemert, D., Spector, S. A., Rouphael, N., Creech, C. B., McGettigan, J., Khetan, S., Segall, N., Solis, J., Brosz, A., Fierro, C., Schwartz, H., Neuzil, K., Corey, L., … Zaks, T. (2021). Efficacy and Safety of the mRNA-1273 SARS-CoV-2 Vaccine. *The New England Journal of Medicine*, *384*(5), 403–416. https://doi.org/10.1056/NEJMoa2035389

Chu, L., McPhee, R., Huang, W., Bennett, H., Pajon, R., Nestorova, B., & Leav, B. (2021). A preliminary report of a randomized controlled phase 2 trial of the safety and immunogenicity of mRNA-1273 SARS-CoV-2 vaccine. *Vaccine*, *39*, 2791–2799. https://doi.org/10.1016/j.vaccine.2021.02.007

Goepfert, P. A., Fu, B., Chabanon, A.-L., Bonaparte, M. I., Davis, M. G., Essink, B. J., Frank, I., Haney, O., Janosczyk, H., Keefer, M. C., Koutsoukos, M., Kimmel, M. A., Masotti, R., Savarino, S. J., Schuerman, L., Schwartz, H., Sher, L. D., Smith, J., Tavares-Da-Silva, F., … De Bruyn, G. (2021). Safety and immunogenicity of SARS-CoV-2 recombinant protein vaccine formulations in healthy adults: interim results of a randomised , placebo-controlled , phase 1 – 2 , dose-ranging study. *The Lancet Infectious Diseases*. https://doi.org/10.1016/S1473-3099(21)00147-X

Heath, P. T., Galiza, E. P., Baxter, D. N., Boffito, M., Browne, D., Burns, F., Chadwick, D. R., Clark, R., Cosgrove, C., Galloway, J., Goodman, A. L., Heer, A., Higham, A., Iyengar, S., Jamal, A., Jeanes, C., Kalra, P. A., Kyriakidou, C., Mcauley, D. F., … Toback, S. (2021). Safety and Efficacy of NVX-CoV2373 Covid-19 Vaccine. *The New England Journal of Medicine*, 1–12. https://doi.org/10.1056/NEJMoa2107659

Keech, C., Albert, G., Cho, I., Robertson, A., Reed, P., Neal, S., Plested, J. S., Zhu, M., Cloney-Clark, S., Zhou, H., Smith, G., Patel, N., Frieman, M. B., Haupt, R. E., Logue, J., McGrath, M., Weston, S., Piedra, P. A., Desai, C., … Glenn, G. M. (2020). Phase 1-2 Trial of a SARS-CoV-2 Recombinant Spike Protein Nanoparticle Vaccine. *The New England Journal of Medicine*, *383*(24), 2320–2332. https://doi.org/10.1056/NEJMoa2026920

Li, J., Hui, A., Zhang, X., Yang, Y., Tang, R., Ye, H., Ji, R., Lin, M., Zhu, Z., Türeci, Ö., Lagkadinou, E., Jia, S., Pan, H., Peng, F., Ma, Z., Wu, Z., Guo, X., Shi, Y., Muik, A., … Zhu, F. (2021). Safety and immunogenicity of the SARS-CoV-2 BNT162b1 mRNA vaccine in younger and older Chinese adults: a randomized, placebo-controlled, double-blind phase 1 study. *Nature Medicine*, *27*, 1062–1070. https://doi.org/10.1038/s41591-021-01330-9

Madhi, S. A., Baillie, V., Cutland, C. L., Voysey, M., Koen, A. L., Fairlie, L., Padayachee, S. D., Dheda, K., Barnabas, S. L., Bhorat, Q. E., Briner, C., Kwatra, G., Ahmed, K., Aley, P., Bhikha, S., Bhiman, J. N., Bhorat, A. E., Plessis, J., Esmail, A., … Izu, A. (2021). Efficacy of the ChAdOx1 nCoV-19 Covid-19 Vaccine against the B.1.351 Variant. *The New England Journal of Medicine*. https://doi.org/10.1056/NEJMoa2102214

Polack, F. P., Thomas, S. J., Kitchin, N., Absalon, J., Gurtman, A., Lockhart, S., Perez, J. L., Pérez Marc, G., Moreira, E. D., Zerbini, C., Bailey, R., Swanson, K. A., Roychoudhury, S., Koury, K., Li, P., Kalina, W. V, Cooper, D., Frenck, R. W. J., Hammitt, L. L., … Gruber, W. C. (2020). Safety and Efficacy of the BNT162b2 mRNA Covid-19 Vaccine. *The New England Journal of Medicine*, *383*, 2603–2615. https://doi.org/10.1056/NEJMoa2034577

Richmond, P., Hatchuel, L., Dong, M., Ma, B., Hu, B., Smolenov, I., Li, P., Liang, P., Han,

© 2022 Haas JW et al. *JAMA Network Open*.

H. H., Liang, J., & Clemens, R. (2021). Safety and immunogenicity of S-Trimer (SCB-2019), a protein subunit vaccine candidate for COVID-19 in healthy adults: a phase 1, randomised, double-blind, placebo-controlled trial. *Lancet (London, England), 397*(10275), 682–694. https://doi.org/10.1016/S0140-6736(21)00241-5

Sadoff, J., Le Gars, M., Shukarev, G., Heerwegh, D., Truyers, C., de Groot, A. M., Stoop, J., Tete, S., Van Damme, W., Leroux-Roels, I., Berghmans, P.-J., Kimmel, M., Van Damme, P., de Hoon, J., Smith, W., Stephenson, K. E., De Rosa, S. C., Cohen, K. W., McElrath, M. J., … Schuitemaker, H. (2021). Interim Results of a Phase 1–2a Trial of Ad26.COV2.S Covid-19 Vaccine. *New England Journal of Medicine,* 1–12. https://doi.org/10.1056/nejmoa2034201

Shinde, V., Bhikha, S., Hoosain, Z., Archary, M., Bhorat, Q., Fairlie, L., Lalloo, U., Masilela, M. S. L., Moodley, D., Hanley, S., Fouche, L., Louw, C., Tameris, M., Singh, N., Goga, A., Dheda, K., Grobbelaar, C., Kruger, G., Ganey, N. C., … Madhi, S. A. (2021). Efficacy of NVX-CoV2373 Covid-19 Vaccine against the B.1.351 Variant. *The New England Journal of Medicine*, *384*(20), 1899–1909. https://doi.org/10.1056/NEJMoa2103055

Walsh, E. E., Frenck, R. W. J., Falsey, A. R., Kitchin, N., Absalon, J., Gurtman, A., Lockhart, S., Neuzil, K., Mulligan, M. J., Bailey, R., Swanson, K. A., Li, P., Koury, K., Kalina, W., Cooper, D., Fontes-Garfias, C., Shi, P.-Y., Türeci, Ö., Tompkins, K. R., … Gruber, W. C. (2020). Safety and Immunogenicity of Two RNA-Based Covid-19 Vaccine Candidates. *The New England Journal of Medicine*, *383*(25), 2439–2450. https://doi.org/10.1056/NEJMoa2027906

© 2022 Haas JW et al. *JAMA Network Open*.
